# A Case of Epileptiform Neuralgia

**Published:** 1882-07

**Authors:** Edward P. Davis


### Clinical Reports.

### ARTICLE VII.

A CASE OF EPILEPTIFORM NEURALGIA. From the Records of the Clinic for Nervous Diseases, Rush Medical College, May 26, 1881. Reported by DR. EDWARD P. DAVIS.

The patient, Mr. L. S. B., æt. forty-three, resides in Scott county, Iowa, in a non-malarious and healthful region. His family history shows insanity, epilepsy and phthisis; no history of alcoholism. Patient appears well nourished, and exhibits more than ordinary intelligence. He has worked very steadily at the saddler's trade, but aside from his nervous diseases, he has had no serious illness. When twenty-one years old, he fell overcome when exposed to the sun. Three months afterward he had convulsions, which occurred at daytime once in two weeks or once a month for thirteen years. These were doubtless epileptiform. He then removed to a more quiet locality, and by exemption from excitement and the use of remedies, his epileptic fits gradually ceased, but the present neuralgia supervened about a year after the cessation of his fits. He has had but one or two convulsions since the neuralgia began. His first neuralgic pain was felt in the first molar of the right upper jaw; this pain returned every five minutes, and did not yield to quinine. The pain grew worse, extending around the face and into the eye; at first coming on only in winter, then in both winter and summer. At present the pain is paroxysmal; the face twitches about the lips; a burning sensation is felt, and great pain over the second division of the fifth nerve. The patient remains conscious, and presses with one hand on the occiput, while with a handkerchief in the other hand he rubs the right side of the face. This rubbing is the result of experiment, while the occipital pressure is involuntary. Pupils are equal; the cornea clear; the conjunctivæ reddened. Pain often radiates over all the branches of the fifth pair; the malar bone is yielding to the patient's pressure. Sight is normal; the tongue is protruded well; smell is normal, but an annoying discharge of mucus persists from the right nostril. Taste, hearing and sensation are normal. Pressure over the supra-orbital foramen elicits slight pain. Pressure over the remaining foramina of exit of the fifth pair gives no pain, but a paroxysm may be elicited by pressure near the zygoma. The pain does not vary in intensity by day or night. Perspiration on the facial region is normal; a feeling of heat is present in the affected side. The patient has exhausted opium, gelsemium, quinine, iron; a combination of chloral hydrate and opium affords partial relief. In the failure of medication, an operation is advised, and on May 28, 1881, Dr. C. T. Parkes proceeded as follows:

Under antiseptic precautions, a curved incision was made over the malar bone, an entrance effected into the antrum of Highmore by a trephine; the rear wall of the antrum was perforated, the floor of the orbit was entered from the spheno-palatine fossa, where the infra-orbital nerve was reached, and followed to the foramen rotundum, where the second division of the fifth was found. An effort was made to stretch the nerve, when it broke, and the contents of the spheno-palatine fossa were removed, including Meckel's ganglion. The second division of the fifth was cut off at the foramen rotundum, and an anterior section was also made, which removed one and one-half inches in all. During the first night hæmorrhage from the nose and mouth occurred, but was not serious. Patient had pain radiating through the remainder of the fifth pair during the second day; twitching of the facial muscles also persisted a few days, but aside from these brief sufferings, and slight soreness about the wound, he described his relief as perfect. The wound healed by first intention, and in six days after the operation the patient went to his home.

Through correspondence, it was learned that his relief persisted until late in the past winter, when the pain recurred in the inferior dental nerve and about the eye. He was advised to visit the city again, and presented himself for examination. An operation upon the inferior dental was recommended, and Prof. Gunn, at the surgical clinic, April 22, trephined through the ramus of the lower jaw, raised the nerve, and excised a quarter of an inch of its extent. The patient made a fair recovery from the operation, but was annoyed by very severe pains for about three weeks. Hypodermic injections of morphia and atropia relieved this pain, and as the wound healed he found relief without further medication. He is now free from pain, but notes uneasy sensations attendant upon cold and damp weather.

The peculiar origin and distribution of the trigeminus, its resemblance to a spinal nerve in the possession of a ganglion upon its posterior root, and its liability to disease, have rendered it an object of great interest to students in all departments of medicine. Recalling its origin, we find it to be from the outer portion of the floor of the fourth ventricle and from the continuation of the posterior horns of gray matter of the cord known as the tubercle of Rolando; the fibers of origin of the motor trunk are traced to the olivary bodies, to an origin common with fibers of the facial. Its sensory distribution will be remembered as embracing the face and fore part of the head, the eye, nose, ear and mouth. Without attempting to discuss the part played by the chorda tympani in the nerve supply of the anterior portion of the tongue, we may assign its innervation to the fifth pair (Quain). The motor distribution of the nerve in question is principally to the muscles of mastication.

In function, the fifth resembles a spinal nerve in the conveyance of both sensory and motor impulses. Its trophic fibers take origin in the Gasserian ganglion, and their function has been strikingly illustrated by the experiments of Samuels, Meissner and Schiff. Cases of disease of the fifth pair, and autopsies revealing alteration of the structure of the ganglion, furnish corroborative evidence of its trophic function. We have reason to believe that the fifth possesses recurrent fibers from clinical observation, the phenomena of the painful points of Varleix being of common occurrence. In view of the extensive distribution of this nerve upon a cutaneous surface exposed without protection to sudden changes of temperature, and its connection with the teeth, so liable to disease, trifacial neuralgia is but a natural sequence of any predisposing malnutrition, inherited or acquired. Thus, Romberg states that it occurs only in advanced life, when the balance of waste and supply is in favor of the former. While the pathology of neuralgia presents much obscurity, we may adduce certain considerations which afford a reasonable, if not a proven explanation.

Healthy nervous action may be regarded as regularity of isomeric activity in the nerve trunk or ganglion in question, constituting the so-called normal tonic influence of the nervous system, this being independent of volition or peripheral impact. A natural analogy would be the condition of magnetism in any body. Any disturbance of molecular equilibrium will result in the interruption of this activity. Such continued disturbance we are accustomed to designate mal-nutrition. Long continued irregularities of nervous force lead to excessive or perverted activities, and the sensation of pain results. In the case described, this was carried to the extreme; the patient's paroxysms came on at varying intervals, but with great suddenness and severity. His natural impulse was to press with all his force over the affected nerves, and this pressure was the result of experiment, and was his only means of mitigating his sufferings, pressure evidently checking the activities of his nerves, as impact checks the vibrations of a sonorous body. Examination of excised neuralgic nerves gives varying results. Lesions found are those of altered nutrition.

The treatment of epileptiform neuralgia has embraced the full extent of anodyne medication, and has included surgical procedure as a last resort. Trousseau has used large doses of opium, finding great tolerance of the drug; while he does not claim to have cured a case, he has relieved many.

Bastian (*British Medical Journal*, June, 1879) cured a case of reflex epileptiform neuralgia by large doses of opium, twenty-five grs. per diem, diminishing the dose without the patient's knowledge. Remack reports a cure of tic doloreux by electricity, while Meyer accomplished this result by the same agent, making especial applications to the cervical sympathetic. Beard and Rockwell report failure after a brief trial in one case, while in case of a man æt. sixty, temperate and of fine physique, eighteen applications of Faradaic electricity cured, the whole duration of the disease being two years. Three cases of severe trifacial neuralgia, not epileptiform, were improved, but not cured, by applications of Faradaic electricity. Hamilton cites a case where clonic spasms existed, the eyes being injected, flow of saliva excessive, gums tender, and points painful on pressure, where no relief was obtained except by large doses of alcohol. Nerve-stretching and resection have been brought forward as perhaps the only directly relieving agents known in these cases, and with results somewhat as follows: For the various neuralgiæ Wagner cites 135 cases, whose results show nine failures; six deaths; thirty-two relapses after several months; twenty relapses up to three years from operation; eighteen cases with no relapses for several months; twenty-three with no relapses after several years; twenty-six cases history after operation unknown. In "Select Topics of Modern Surgery," by Drs. Fenger and Lee, of Chicago, twelve cases are cited wherein stretching of the fifth pair resulted in nine recoveries; one failure; one partial relief; one relapse. An operation upon the infra-orbital nerve for neuralgia of the orbital branches of the fifth pair, by Dr. Bogue, of Chicago, resulted in six months relief, and a severe relapse. Nussbaum and Patonbau advocate the ligation of the carotid artery for general neuralgiæ of the fifth pair, while neurotomy is commended in localized affections; it has been observed that section of the inferior dental nerve is peculiarly successful when union does not occur.

The recent experiments of M. Weit (*Progrès Médicalè*, March 4, 1882) demonstrate the influence of nerve-stretching upon the centers themselves, tension of the pneumogastric having produced an injection of the medulla visible to the unaided eye. It will have been observed in the case described, that points painful upon pressure were not as abundant as in many cases. The absence of this irritation of the recurrent nerves would indicate a neuralgia of central origin; a point which the preceding epilepsy of the patient proves correct.

